# The Influence of Angiotensin Converting Enzyme and Angiotensinogen Gene Polymorphisms on Hypertrophic Cardiomyopathy

**DOI:** 10.1371/journal.pone.0077030

**Published:** 2013-10-25

**Authors:** Rong Luo, Xiaoping Li, Yuequn Wang, Yongqing Li, Yun Deng, Yongqi Wan, Zhigang Jiang, Wei Hua, Xiushan Wu

**Affiliations:** 1 The Center for Heart Development, Key Laboratory of MOE for Developmental Biology and Protein Chemistry, College of Life Sciences, Hunan Normal University, Changsha, Hunan, P.R. China; 2 Medical Science Research Center, Guangxi Medical University, Nanning, Guangxi, P.R China; 3 Cardiac Arrhythmia Center, Cardiovascular Institute and Fuwai Hospital, Chinese Academy of Medical Sciences, Peking Union Medical College, Beijing, P.R China; Max-Delbrück Center for Molecular Medicine (MDC), Germany

## Abstract

Some studies have reported that angiotensin converting enzyme (ACE) and angiotensinogen (AGT) genes have been associated with hypertrophic cardiomyopathy (HCM). However, there have been inconsonant results among different studies. To clarify the influence of ACE and AGT on HCM, a systemic review and meta-analysis of case-control studies were performed. The following databases were searched to indentify related studies: PubMed database, the Embase database, the Cochrane Central Register of Controlled Trials database, China National Knowledge Information database, and Chinese Scientific and Technological Journal database. Search terms included “hypertrophic cardiomyopathy”, “angiotensin converting enzyme” (ACE) or “ACE” and “polymorphism or mutation”. For the association of AGT M235T polymorphism and HCM, “angiotensin converting enzyme” or “ACE” was replaced with “angiotensinogen”. A total of seventeen studies were included in our review. For the association of ACE I/D polymorphism and HCM, eleven literatures were included in the meta-analysis on association of penetrance and genotype. Similarly, six case-control studies were included in the meta-analysis for AGT M235T. For ACE I/D polymorphism, the comparison of DI/II genotype vs DD genotype was performed in the present meta-analysis. The OR was 0.73 (95% CI: 0.527, 0.998, *P* = 0.049, power = 94%, alpha = 0.05) after the study which deviated from Hardy-Weinberg Equilibrium was excluded, indicating that the ACE I/D gene polymorphism might be associated with HCM. The AGT M235T polymorphism did not significantly affect the risk of HCM. In addition, ACE I/D gene polymorphism did not significantly influence the interventricular septal thickness in HCM patients. In conclusion, the ACE I/D polymorphism might be associated with the risk of HCM.

## Introduction

Hypertrophic cardiomyopathy (HCM) is characterized by left ventricular hypertrophy (LVH) with predominant involvement of the interventricular septum (IVS) in the absence of hypertension, valvular heart disease, or other evident cardiac or systemic cause [1], and microscopically by cardiomyocyte hypertrophy, myofibrillar disarray, and fibrosis [2]. HCM has a wide spectrum of clinical presentations ranging from asymptomatic hypertrophy to refractory heart failure and sudden cardiac death. The prevalence of HCM is estimated to be around 1/500 in the general population [3] carrying an annual cardiovascular mortality rate of 0.7–1.4% [4]. Sudden death is the most common mode of death with an overall annual mortality rate about 1% [5].

HCM is frequently caused by mutations in genes encoding sarcomeric proteins [6], [7], [8]. To date, more than 13 gene mutations coding for sarcomeric proteins have been found in patients with HCM [9]. Many genes responsible for HCM remain to be identified. However, phenotypic expression and clinical course vary considerably [8], and some family members even fail to express the disease although the same identical mutation is seen in them, indicating that the disease course is not solely dependent on the pathogenic gene. Other factors, such as the additional modifier genes or environmental influences might also influence disease susceptibility [10]. It is reported that several gene polymorphisms, including those encoding the components of the renin-angiotensin system (RAS), have been associated with the risk of developing LVH, and could also modify the clinical phenotype in HCM patients [11], [12], [13], [14], [15]. It is reported that RAS acted on cellular hypertrophy and cell proliferation [16], and therefore played a regulatory role in cardiac function, blood pressure, and electrolyte homeostasis [17]. In the end, it can affect both left cardiac ventricle (LV) hypertrophy and remodeling [14].

The deletion/insertion (D/I) polymorphism in intron 16 of the gene encoding angiotensin converting enzyme (ACE) on chromosome 17q23 has been associated with several cardiovascular disorders including LVH in untreated hypertension, complications of atherosclerosis [14], and HCM [15], [18], [19], [20], [21], [22]. Angiotensinogen (AGT) gene, located at chromosome 1q4, has a polymorphism at position codon 235 with threonine instead of methionine (M235T).. It is reported that there were linkages between AGT M235T polymorphism and several cardiovascular diseases such as myocardial infarction, LVH and coronary atherosclerosis [23]. Moreover, the TT genotype of AGT might be a genetic marker of electrocardiographically determined LVH since a positive association has been reported between AGT M235T polymorphism and HCM [24].

In addition, indices of cardiac hypertrophy, such as the mean interventricular septal thickness (IVST), the mean left ventricular mass index, were greater in HCM patients with the ACE DD genotype as compared with II genotype [1], [25], [26].

In spite of the above mentioned reports associating RAS and HCM, the studies from different populations have been conflicting and the role of the RAS system in modifying the phenotype in HCM remains controversial. As meta-analysis is a reliable way to combine information from many studies and thus may provide more conclusive answers, we decided to evaluate the influence of ACE and AGT polymorphisms on the HCM phenotype.

## Methods

### Search strategy

The following database were searched: PubMed database, the Embase database, the Cochrane Central Register of Controlled Trials database, China National Knowledge Information database, and the Chinese Scientific and Technological Journal database were searched.

For the association of ACE I/D and HCM, the following search term were used in searching the PubMed database: “hypertrophic cardiomyopathy”, “angiotensin converting enzyme” or “ACE” and “polymorphism or mutation”. Alternatively, “angiotensin converting enzyme” or “ACE” was replaced with “angiotensinogen” for the association of M235T genotype and HCM. The last search was up to July 2013. No language is limited. In addition, the references of retrieved articles were also screened to find the related papers.

### Study selection

Two investigators independently reviewed all studies and extracted the data using a standard information extraction and reached consensus on all items. Only those articles that detected the genotype polymorphism and the patients were diagnosed as HCM were included. For the meta-analysis for the association of genotype and penetrance of HCM, only studies in which control group were healthy people were included. In the present study, we also performed a meta-analysis on the association of IVST/maximal left ventricle wall thickness (MWT) and ACE genotype polymorphism, which studies were all case-case studies.

### Risk of bias and data extraction

For the meta-analysis for the association of genotype polymorphism and penetrance of HCM, all studies were case-control studies. The quality of studies was independently assessed by two reviewers using a risk of bias assessment for genetic association studies [27], [28]. The following data were collected: the first author of studies, year of publication, number of genotypes in cases and controls, the *P* value of Hardy-Weinberg equilibrium (HWE) in control, origin of control subjects, mean age in case and controls. With regard to risk of bias, each item was classified as yes, no, unclear, which refer to low risk, high risk, and unclear if insufficient information was available for assessment [27], [28].

### Statistical analysis

For the meta-analysis for the association of genotype and penetrance of HCM, the statistical analysis was performed according to the previous studies [27], [29], [30], [31]. Briefly, HWE in the control group was tested with the exact test in every included study. Then, a mixed-effects hierarchical model with a logit link function was applied to gauge whether the overall gene effect was significant using the xtmelogit command in Stata software to test the likelihood ratio (LR) [27], [30], [31]. If the overall gene effect was statistically significant, further comparisons of odds ratios (ORs): OR_1_ (II vs DD for ACE I/D polymorphism; MM vs TT for M235T); OR_2_ (DI vs DD for ACE I/D allele; MT vs TT for AGT M235T); and OR_3_ (II vs DI for ACE I/D allele; MM vs MT for M235T) were explored. The heterogeneity among different studies was tested by the chi-square-based Q statistic test and I square statistics. *P*<0.1 [32] rather than 0.05 was considered significant heterogeneity for the chi-square-based Q testing and I^2^<25% is considered low heterogeneity among studies; 25% to 50% moderate, and >50% as high level heterogeneity [32]. If heterogeneity was significant, the results were pooled using a random effect model and the inverse variance method [33]. Otherwise, the fix-effect model was used.

The most appropriate genetic models were selected as follows [30]:

- Dominant model if OR_1_ = OR_2_≠1 and OR_3_ = 1;
- Recessive model if OR_1_ = OR_3_≠1 and OR_2_ = 1;
- Overdominant model if OR_2_ = 1/OR_3_≠1 and OR_1_ = 1;
- Codominant model if OR_1_>OR_2_>1 and OR_1_>OR_3_>1 (or OR_1_<OR_2_<1 and OR_1_<OR_3_<1).

Finally, once the appropriate genetic model was identified, results were pooled again under this genetic model. The significance of the pooled OR was determined using the Z-test. *P*<0.05 was considered statistically significant.

As for the meta-analysis for the association of thickness of IVST/MWT and genotype polymorphism, the combined standard mean difference was compared.

Egger's test and the visual symmetry of funnel plot were assessed to examine publication bias of the related studies.

In addition, meta-regression was used to explore the source of the heterogeneity by the Stata software. Sensitivity analysis was also performed to test the robustness of the results by excluding studies that deviated from HWE. The Stata software (version 12.0; Stata Corporation College Station, Texas) and Review Manager software 5.2 (Cochrane Collaboration, http://ims.cochrane.org/revman/download) was used to performed the present meta-analysis. Furthermore, the PS software [34] (version 3.0, http://biostat.mc.vanderbilt.edu/wiki/Main/PowerSampleSize) was used to calculate the power of statistical test.

## Results

### Search results

**Figure 1:**
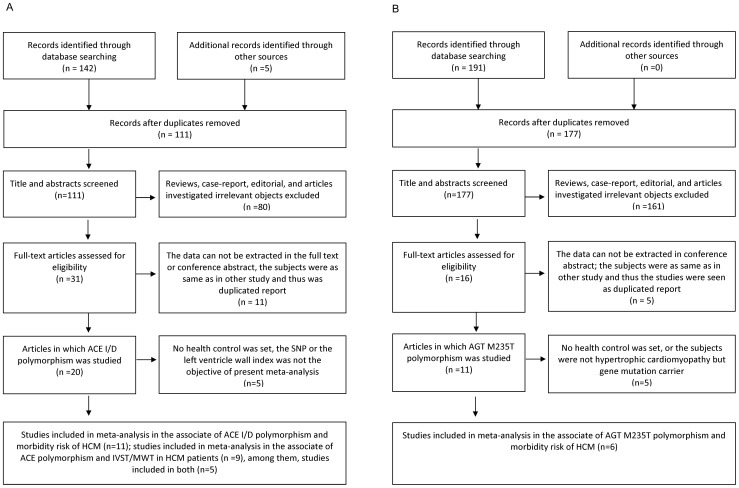
Flow diagram of studies selection. IVST, interventricular septum thickness; SNP, Single Nucleotide Polymorphism. HCM, hypertrophic cardiomyopathy; ACE, angiotensin converting enzyme; AGT, angiotensinogen. Fig. 1a, ACE I/D flow diagram; Fig. 1b, AGT flow diagram.

A total of seventeen studies were included in our review [1], [13], [20], [21], [23], [25], [26], [35], [36], [37], [38], [39], [40], [41], [42], [43], [44]. For ACE I/D polymorphism, a total of 31 prospective trials surveyed the polymorphism of ACE I/D. Of these, the data can not be extracted in full text or conference abstract in ten studies, either no healthy control were set or the single nucleotide polymorphism, or the IVST was not the objective of present meta-analysis in five studies. The subjects in one study [15] were as same as in other study [23] and thus the study was duplicated reports. In the end, eleven literatures were included in the meta-analysis on association of penetrance and genotype polymorphism. Similarly, for the association of penetrance and genotype, six studies were included in the meta-analysis for AGT M235T. Meanwhile, as for the association of IVST/MWT and genotype, nine studies were included in the present meta-analysis for ACE I/D. We did not perform a meta-analysis on the relation of IVST and M235T genotype due to the insufficient number of studies. Figure 1 showed the flow diagram of studies selection. Table 1 and Table 2 lists the eligible studies which were included in the association of penetrance and genotype for ACE I/D and AGT M235T, respectively.

**Table 1:** Characteristics of eligible studies in the meta-analysis (ACE I/D).

		HCM				Control							
		Genotypes				Genotypes				HWE in control			
First author, Year	Country	N	II	ID	DD	N	II	ID	DD	*P* value	Control subjects	Mean age in Case (years)	Mean age in Control (years)
Kawaguchi 2003	Japan	80	26	41	13	88	43	28	17	0.0045	Unaffected siblings and children	/	/
Coto 2010	Spain	207	35	100	72	300	46	135	119	0.4527	Ethnic-matched (Caucasian)	/	51±17
Yamada 1997	Japan	71	31	32	8	122	50	55	17	0.7640	Healthy individuals	59.1±10.3	60.2±11.0
Marian 1993	USA	100	7	49	44	106	22	46	38	0.2495	Normal relatives	/	/
Pfeufer 1996	Germany	50	26	24	50	36			14	Yes	Age and gender matched unrelated healthy subjects	55±15	48±17
Kaya 2010	Turkey	63	8	34	21	20	5	9	6	0.6620	Ethic-matched healthy controls	55.94±14.8	53.9±7.9
Rai 2008	India	118	11	63	44	164	47	87	30	0.3532	Healthy,age,sex,and ethnicity matched controls	/	/
Ogimoto 2002	Japan	138	53	64	21	205	83	95	27	0.9821	Healthy Japanese	63±13	70±9
Doolan 2004	Austrilia	36	10	14	12	200	48	94	58	0.4150	Age and gender matched control population	49.17±20.56	/
Moiseev 1997	Russia	13	2	5	6	168	33	55	80	0.2445	Normal subjects	31.5±9.1	45.4±11.5
Lopez-Haldon 1999	Spain	40	2	13	25	269	33	125	111	0.8097	Healthy subjects	44.3±15.9	/

Note, HCM; hypertrophic cardiomyopathy; HWE, Hardy Weinberg Equilibrium.

**Table 2:** Characteristics of eligible studies in the meta-analysis (AGT M235T).

		HCM				Control							
		Genotypes				Genotypes				HWE in control			
First author, Year	Country	N	TT	MT	MM	N	TT	MT	MM	*P* value	Control subjects	Mean age in Case (years)	Mean age in Control (years)
Kawaguchi 2003	Japan	96	67	28	1	160	94	61	5	0.1877	Healthy subjects	/	/
Rao 2011	India	150	70	68	12	165	65	85	15	0.0841	Age and sex matched healthy subjects, blood donors	52.6±8.5	50.44±9.2
Yamada 1997	Japan	71	37	29	5	122	76	44	2	0.1190	Healthy individuals	59.1±10.3	60.2±11.0
Coto 2010	Spain	205	41	100	64	300	60	145	95	0.7291	Ethnic matched, Healthy individuals did not have symptoms of cardiovascular disease	/	51±17
Cai 2004	China	72	45	22	5	80	36	30	14	0.0916	Healthy control	51.7±16.3	53.3±18.4
Lopez-Haldon 1999	Spain	40	7	20	13	269	54	128	87	0.5795	Healthy subjects	44.3±15.9	/

Note, HCM; hypertrophic cardiomyopathy; HWE, Hardy Weinberg Equilibrium.

### The quality of studies

As described in Table 3, all included studies for association of penetrance and genotype polymorphism were clearly ascertained of HCM and some causes that might lead to myocardial hypertrophy were excluded. All genotype tests were assayed using the polymerase chain reaction (PCR) in all studies. Neither genotyping error rate nor genotype testing under blind condition was described in any study. There was no selective reporting in all including studies and eight studies mentioned the HWE in control group [1], [21], [23], [25], [36], [39], [40], [42].

**Table 3:** Determination of risk assessment bias by included studies of meta-analysis.

First Author, Year	Ascertainment of HCM	Exclusion	Ascertainment of Control	Quality Control for Genotyping	Population Stratification	Confounding Bias	Selective reporting	HWE
Yamada 1997	Electrocardiogram, chest x-ray, echocardiography, left ventriculography, coronary angiography and biopsies	Hypertension, ischemic heart disease, valvular heart disease, congenital malformations, intrinsic pulmonary, renal or metabolic disease	Medical checkup and did not exhibit any serious disorders.	Unclear	Unclear	Yes	Yes	Yes
Kawaguchi 2003(ACE)	Physical examination, including coronary angiography and cardiac biopsy. Echocardiography	Myocardial infarction, hypertension, thyroid disease and other metabolic disease that may cause left ventricular hypertrophy were excluded	Did not have HCM Echocardiographically	Unclear	Yes	Unclear	Yes	Yes
Kawaguchi 2003(AGT M235T)	Echocardiography	Myocardial infarction, hypertension, thyroid disease and other metabolic disease that may cause left ventricular hypertrophy were excluded	Healthy subjects without known hypertension and LVH matched by age and sex.	Unclear	Yes	Yes	Yes	Yes
Kaya 2010	Echocardiography	Excluded demonstrable hypertrophic stimulus such as hypertension or aortic stenosis.	Ethnic matched healthy controls	Unclear	Yes	Yes	Yes	Yes
Coto 2010	Echocardiography	Excluded hypertension, valvular disease, and myocardial infarction	Ethnic matched, excluded the existence of cardiac diseases.	Unclear	Yes	Unclear	Yes	No
Rao 2011	Echocardiography	Thyroid disease, hypertension and myocardial infarction were excluded	Age and sex matched healthy subjects, blood donors.	Unclear	Yes	Yes	Yes	No
Rai 2008	Echocardiography	Identified unexplained left ventricular hypertrophy	Healthy, age, sex and ethnicity matched controls.	Unclear	Yes	Yes	Yes	Yes
Marian 1993	detailed cardiovascular examination and two-dimensional echocardiography.	Familial patients which excluded hypertension or other potential causes of the hypertrophy	Normal relatives	Unclear	Unclear	Unclear	Yes	No
Ishanov 1998	Echocardiography	Myocardial infarction, hypertension, thyroid disease, and other metabolic disease were excluded	Age and sex matched without known hypertension and left ventricular hypertrophy	Unclear	Yes	Yes	Yes	No
Ogimoto 2002	M-mode and two-dimensional echocardiography	Other causes for left ventricular hypertrophy, patients had undergone cardiac surgery	Who were free of any history or symptoms of cardiovascular disease and not taking any medications	Unclear	Yes	Unclear	Yes	Yes
Lopez-Haldon 1999	Doppler echocardiography	Age less than 18 years, existence of other cause of myocardial hypertrophy (hypertension, valve disease, or presence of poor echocardiographic image)	Normal subjects	Unclear	Unclear	Unclear	Yes	Yes
Moiseev 1997	Echocardiography	Essential hypertension, myocardial infarction	Normal subjects	Unclear	Yes	Yes	Yes	No
Doolan 2004	Family history, electrocardiographic criteria	Hypertension	Normal control	Unclear	Yes	Yes	Yes	Yes
Cai 2004	Echocardiography	Hypertension, coronary heart disease, valvular heart disease	Healthy control who were free of cardiovascular and pulmonary vascular disease	Unclear	Yes	Yes	Yes	Yes

Note, HCM; hypertrophic cardiomyopathy; HWE, Hardy Weinberg Equilibrium.

### HWE and allele frequencies

HWE in control were showed in Table 1 and Table 2. Of 17 studies, one study in control was deviated from HWE. The minor allele in control group was I allele and M allele in ACE I/D and AGT M235T, respectively. There was heterogeneity among the 10 studies for ACE I/D (χ^2^ = 170.945 (9 df), *P*<0.01) except for one study in which “I” allele frequency can not be calculated [44] and the pooled I allele frequency using the random effects model was 49.39 percent (95% CI: 41.01, 57.77). Similarly, there was heterogeneity in the minor allele among studies for AGT M235T (*P*<0.01).

### Meta-analysis of association of genotype and HCM phenotype

For the association of genotype and penetrance, the eligible 11 studies included 916 cases and 1692 healthy people for ACE I/D were brought into the meta-analysis. The overall gene effect was significant [LR = 22.49 (df = 2), *P*<0.01]. The OR_1_ (II vs DD) and OR_2_ (DI vs DD) was 0.631 (95% CI: 0.344, 1.158), 0.695 (95%CI: 0.522, 0.924), respectively. The ORs were significantly heterogeneous for OR_1_ [χ^2^ = 25.93 (9 df), *P* for heterogeneity <0.01, I^2^ = 65.3%]. Neither the publishing year nor the region was the main origin of the heterogeneity. The meta-regression did not identify the source of heterogeneity. Therefore, the random effects model was used to pool these studies by logistic regression. The gene model was most likely to be dominant model. Then, the comparison of DI+II vs DD was performed in the present meta-analysis.

**Figure 2:**
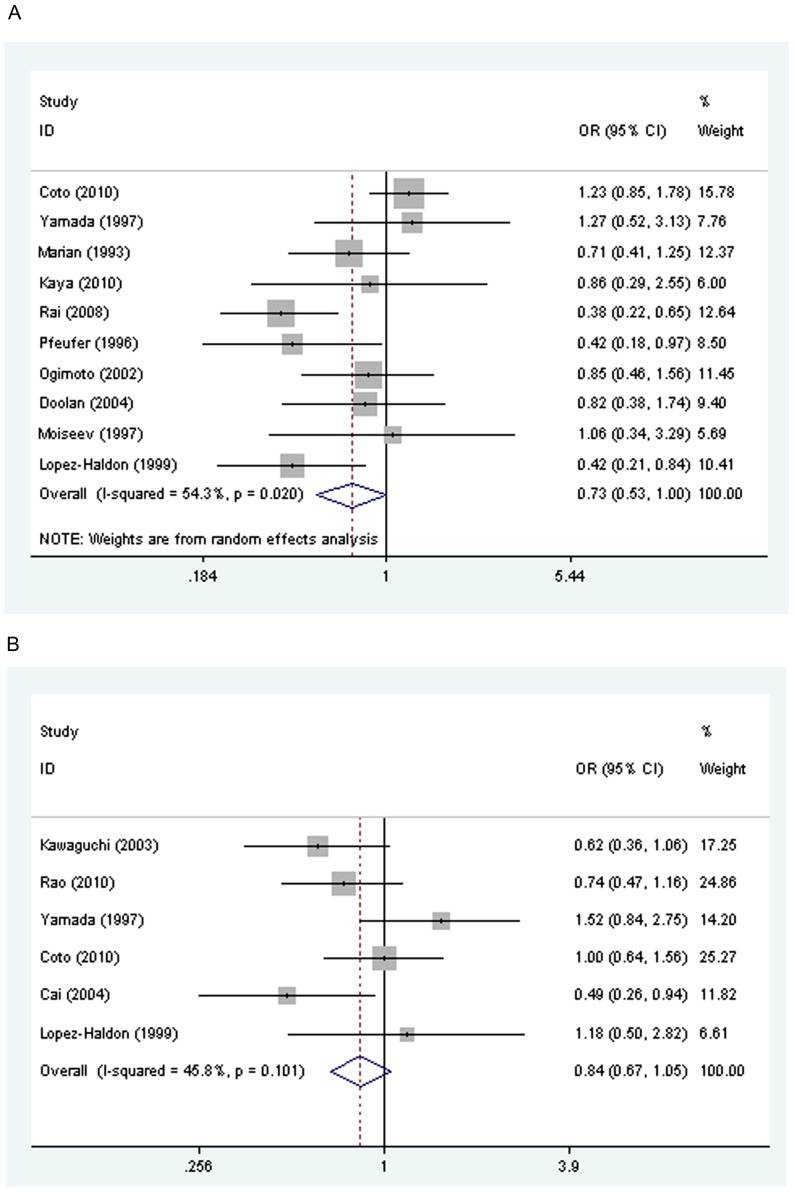
Meta-analysis of the association between ACE I/D and AGT M235T polymorphisms and HCM penetrance. OR in Fig. 2a indicated that the OR of DI/II vs DD. The pooled OR was 0.73 (95 CI: 0.527, 0.998, *P* = 0.049). Similarly, OR indicated that the OR of MM/MT vs TT in Fig. 2b. HCM, Hypertrophic cardiomyopathy. Fig. 2a, ACE I/D; Fig. 2b, AGT M235T.

OR under indicating genetic model was 0.757 (95% CI: 0.56, 1.02, *P* = 0.071), sensitivity analysis indicated that the OR was 0.73 (95% CI: 0.527, 0.998, *P* = 0.049, Fig. 2a, power = 94%, alpha = 0.05) after the study [23] which is deviated from HWE was excluded, demonstrating that ACE “DI/II” genotype was lower in HCM patients than the control group, suggesting that ACE I allele might protect human from HCM.

Publication bias for the association of ACE I/D polymorphism and HCM penetrance is unlikely by using a funnel plot which symmetrical (in Egger's test *P* = 0.603).

As for the association of genotype and penetrance of AGT M235T, six studies consisted of 634 cases and 1096 healthy people were included. The overall gene effect was not significant [LR = 2.68 (df = 2), *P* = 0.26]. Even so, the most likely dominant model also used to estimated the OR (MT+MM vs TT for M235T, OR = 0.84, 95% CI: 0.68, 1.054; *P* = 0.13; Fig. 2b, power = 39.8%, alpha = 0.05). The power of statistical test demonstrates that we are able to reject the null hypothesis that this odds ratio equals 1 with probability (power) 39.8%. All these results indicated that 235 codon transition in AGT did not affect significantly the risk of HCM. The heterogeneity among the included 6 studies is not significant [χ^2^ = 9.22 (5df), *P* for heterogeneity = 0.101, I^2^ = 45.8%]

Sensitivity analysis indicated that excluding the study by Yamada et al [1] could reverse the statistical significance (OR, 0.76; 95% CI: 0.60, 0.97). However, there is no rational reason to exclude this study. A symmetrical funnel plot and Egger's test indicated that publication bias is unlikely for AGT M235T polymorphism and HCM penetrance (*P* = 0.863).

### Meta-analysis of association of IVST/MWT and genotype in HCM patients

**Figure 3:**
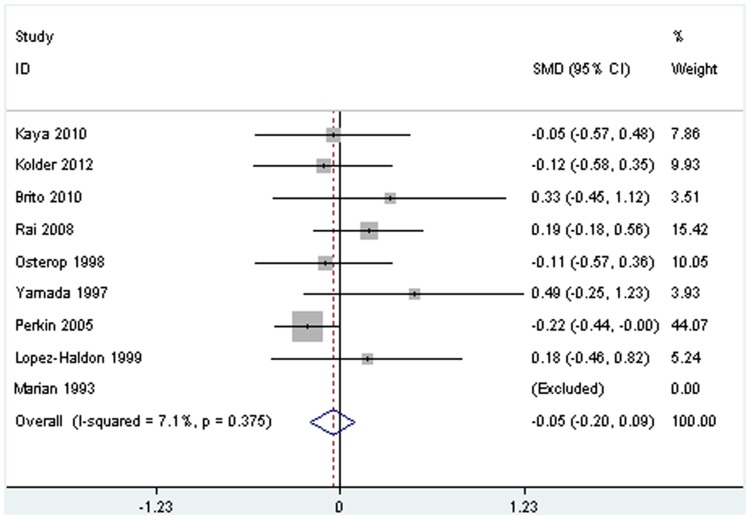
Meta-analysis of the association between ACE I/D polymorphism and IVST/MWT in HCM patients. HCM, hypertrophic cardiomyopathy; IVST, interventricular septum thickness; MWT, maximal left ventricle wall thickness. The SMD represents the standard mean difference of IVST/MWT (mm) between DI/II and DD genotype in HCM patients.

For the association of IVST/MWT and ACE I/D genotype in HCM patients, there were eight studies included in the present meta-analysis. The pooled standard mean difference was 0.05 (95%CI:−0.20, 0.09, *P* = 0.47, Fig. 3, power = 29.8%, alpha = 0.05), demonstrating that we are able to reject the null hypothesis that the IVST/MWT means of the DI/II genotype and DD genotype in HCM patients are equal with probability (power) 29.8%, indicating that there is no significant different in the IVST/MWT between DI/II and DD genotype. The heterogeneity among the included 8 studies is not significant [χ^2^ = 7.54 (7 df), *P* for heterogeneity = 0.375, I^2^ = 7.1%].

Sensitivity analysis indicated that the pooled IVST standard mean difference was not statistically significant (standard mean difference:−0.09; 95% CI: −0.31, 0.14; *P* = 0.44) after excluding the three studies which the left ventricular wall index was MWT rather than IVST. Similarly, there is no publication bias for the association of ACE I/D genotype and IVST/MWT because a symmetrical funnel plot.

## Discussion

The present meta-analysis manifested that frequency of the *ACE* DI/II genotype was lower in HCM patients than the normal controls after excluding the study which did not observe the HWE, indicating that ACE I/D polymorphism might be associated with the risk of HCM, with “I” allele at ACE 16 exon might have a protective effect from HCM. That is, DD genotype might be a risk factor for HCM.

HCM is a primary disorder without pressure overload and has been thought to be genetically heterogeneous [15]. HCM can be considered a polygenic disease with different degrees of penetrance and mutations. Several genes including those encoding the components of the RAS have emerged as the potential modifier in HCM [14], [15], [45]. In RAS, renin catalyses the cleavage of AGT to the decapeptide angiotensin I [46], which is further converted into angiotensin II by ACE catalyzing, the biologically vasoconstrictive peptide of the RAS. Angiotensin II has various effects including hypertrophic, and possibly hyperplastic, effects on vascular smooth muscle cells and cardiomyocytes, and increases extracellular collagen matrix synthesis. The potent myotrophic action of angiotensin II makes it likely that the cardiac RAS plays a role in the development of cardiac hypertrophy. Angiotensin II exerts most of its known cellular actions through the AT1R [47].

It is reported that ACE inhibitors can reduce the synthesis of angiotensin II and attenuate such cardiac hypertrophy pathophysiological processes [48]. ACE is 21 kb length, including 26 exons, located on long arm of chromosome 17 (17q23.3) locus of the human genome. It will be inherited independently of the diseased sarcomeric genes which are located on different chromosomes. The restriction fragment length polymorphism, a 287 base pair (bp) insertion/deletion (I/D), is located inside intron 16 of the ACE gene and corresponds to an Alu repetitive sequence. DD genotype subjects had a higher level of ACE and angiotensin II and, consequently, an increase in hypertrophy and fibrosis [13], [14], [36], [49]. That is, the ACE levels in the human heart are in part determined by the so-called insertion/deletion (I/D) polymorphism [49]. Therefore, the angiotensin II levels increased and then also affect the phenotypic expression in HCM [36]. Moreover, ACE DD genotype influenced LVH in patients with a β-myosin Arg403Leu mutation but did not influence those with other mutation, indicated that gene-gene interactions between causal mutation and modifier genes might influence the disease phenotype and the influence of ACE gene polymorphism might depend on the underlying mutation [25].

It is reported that patients with higher levels of ACE activity had large left ventricular indices [50]. Therefore, indices of cardiac hypertrophy were greater in HCM patients with DD genotype as compared with others [13], [19]. However, in the present meta-analysis, we fail to indicate that the IVST is significant different between DI/II and DD genotype. Apart from the insufficient number of studies and heterogeneity among studies, one of probable causes might be that the influence of RAS polymorphisms may depend on the underlying mutation [22]. Most likely, it is due to the following factor, the age of diagnosis of including subjects, duration of illness, severity of the disease, etc.

In humans, the AGT gene is located on chromosome 1q42 and comprises five exons and four introns spanning 12 kb [51]. A substitution of 704 T>C transition in exon 2, results in a methionine to threonine exchange at codon 235 (M235T), the latter is associated with higher levels of AGT [52]. However, the present meta-analysis indicates that M235T polymorphism of AGT gene is not related to the prevalence of HCM, and sensitivity analysis indicated that excluding one study will change the statistical significance. This might due to relatively insufficient number of studies. This study should preferably include approximately 1726 case patients and 2984 control patients if the ratio of control to case patients remains (power = 80%, alpha = 0.05). The variability of results in HCM may be partly accounted for by the heterogeneous patient populations in most studies since the effect of RAS polymorphisms might be relative small to the effect of the underlying primary aetiological mutation [19]. In addition, AGT M235T nucleotide transition is associated with sporadic hypertrophic cardiomyopathy (SHCM) rather than familial hypertrophic cardiomyopathy (FHCM) in which the mutations in sarcomeric genes have been found to be associated with the disease [37].

The results of this meta-analysis should be interpreted with some degree of caution, because there were several limitations in our analysis. First, we fail to subgroup the FHCM and SHCM in HCM patients. In addition, we also failed to subgroup deferent ethnicities due to the relatively insufficient studies. Taken together, all of these limitations may have affected the results of the present study.

In conclusion, the ACE DI/II genotype might protect human from HCM. More additional trials will be needed to clarify role of ACE I/D and AGT M235T polymorphisms in HCM.

## Supporting Information

Checklist S1 PRISMA Checklist.
(DOC)

Appendix S1 An appendix for Stata commands used in the present meta-analysis.
(DOC)

## References

[1] Yamada Y, Ichihara S, Fujimura T, Yokota M (1997) Lack of association of polymorphisms of the angiotensin converting enzyme and angiotensinogen genes with nonfamilial hypertrophic or dilated cardiomyopathy. Am J Hypertens 10: 921–928. 9270088 10.1016/s0895-7061(97)00112-x

[2] Ommen SR, Nishimura RA (2004) Hypertrophic cardiomyopathy. Curr Probl Cardiol 29: 239–291. 15123994 10.1016/j.cpcardiol.2004.01.001

[3] Orenes-Pinero E, Hernandez-Romero D, Jover E, Valdes M, Lip GY, et al (2011) Impact of polymorphisms in the renin-angiotensin-aldosterone system on hypertrophic cardiomyopathy. J Renin Angiotensin Aldosterone Syst 12: 521–530. 21507890 10.1177/1470320311405247

[4] Marian AJ (2000) Pathogenesis of diverse clinical and pathological phenotypes in hypertrophic cardiomyopathy. Lancet 355: 58–60. 10615904 10.1016/s0140-6736(99)06187-5

[5] Maron BJ (2002) Hypertrophic cardiomyopathy: a systematic review. Jama 287: 1308–1320. 11886323 10.1001/jama.287.10.1308

[6] Marian AJ, Roberts R (1995) Recent advances in the molecular genetics of hypertrophic cardiomyopathy. Circulation 92: 1336–1347. 7648684 10.1161/01.cir.92.5.1336

[7] Niimura H, Bachinski LL, Sangwatanaroj S, Watkins H, Chudley AE, et al (1998) Mutations in the gene for cardiac myosin-binding protein C and late-onset familial hypertrophic cardiomyopathy. N Engl J Med 338: 1248–1257. 9562578 10.1056/NEJM199804303381802

[8] Spirito P, Seidman CE, McKenna WJ, Maron BJ (1997) The management of hypertrophic cardiomyopathy. N Engl J Med 336: 775–785. 9052657 10.1056/NEJM199703133361107

[9] Ho CY, Seidman CE (2006) A contemporary approach to hypertrophic cardiomyopathy. Circulation 113: e858–862. 16785342 10.1161/CIRCULATIONAHA.105.591982

[10] Xue H, Wang H, Wang XJ, Sun K, Wang SX, et al (2010) Atrial natriuretic peptide gene polymorphism is not associated with hypertrophic cardiomyopathy. Chin Med J (Engl) 123: 188–192. 20137368

[11] Bleumink GS, Schut AF, Sturkenboom MC, Deckers JW, van Duijn CM, et al (2004) Genetic polymorphisms and heart failure. Genet Med 6: 465–474. 15545741 10.1097/01.gim.0000144061.70494.95

[12] Keren A, Syrris P, McKenna WJ (2008) Hypertrophic cardiomyopathy: the genetic determinants of clinical disease expression. Nat Clin Pract Cardiovasc Med 5: 158–168. 18227814 10.1038/ncpcardio1110

[13] Perkins MJ, Van Driest SL, Ellsworth EG, Will ML, Gersh BJ, et al (2005) Gene-specific modifying effects of pro-LVH polymorphisms involving the renin-angiotensin-aldosterone system among 389 unrelated patients with hypertrophic cardiomyopathy. Eur Heart J 26: 2457–2462. 16087648 10.1093/eurheartj/ehi438

[14] Wang JG, Staessen JA (2000) Genetic polymorphisms in the renin-angiotensin system: relevance for susceptibility to cardiovascular disease. Eur J Pharmacol 410: 289–302. 11134677 10.1016/s0014-2999(00)00822-0

[15] Yoneya K, Okamoto H, Machida M, Onozuka H, Noguchi M, et al (1995) Angiotensin-converting enzyme gene polymorphism in Japanese patients with hypertrophic cardiomyopathy. Am Heart J 130: 1089–1093. 7484741 10.1016/0002-8703(95)90213-9

[16] Sadoshima J, Izumo S (1993) Molecular characterization of angiotensin II–induced hypertrophy of cardiac myocytes and hyperplasia of cardiac fibroblasts. Critical role of the AT1 receptor subtype. Circ Res 73: 413–423. 8348686 10.1161/01.res.73.3.413

[17] Griendling KK, Murphy TJ, Alexander RW (1993) Molecular biology of the renin-angiotensin system. Circulation 87: 1816–1828. 8389259 10.1161/01.cir.87.6.1816

[18] Lechin M, Quinones MA, Omran A, Hill R, Yu QT, et al (1995) Angiotensin-I converting enzyme genotypes and left ventricular hypertrophy in patients with hypertrophic cardiomyopathy. Circulation 92: 1808–1812. 7671365 10.1161/01.cir.92.7.1808

[19] Ortlepp JR, Vosberg HP, Reith S, Ohme F, Mahon NG, et al (2002) Genetic polymorphisms in the renin-angiotensin-aldosterone system associated with expression of left ventricular hypertrophy in hypertrophic cardiomyopathy: a study of five polymorphic genes in a family with a disease causing mutation in the myosin binding protein C gene. Heart 87: 270–275. 11847170 10.1136/heart.87.3.270 PMC1767035

[20] Marian AJ, Yu QT, Workman R, Greve G, Roberts R (1993) Angiotensin-converting enzyme polymorphism in hypertrophic cardiomyopathy and sudden cardiac death. Lancet 342: 1085–1086. 8105312 10.1016/0140-6736(93)92064-z

[21] Lopez-Haldon J, Garcia-Lozano JR, Martinez Martinez A, Nunez-Roldan A, Burgos Cornejo J (1999) [The effect of polymorphisms of the angiotensin-converting enzyme and angiotensinogen genes on the phenotypic expression of Spanish patients with hypertrophic cardiomyopathy]. Med Clin (Barc) 113: 161–163. 10480137

[22] Tesson F, Dufour C, Moolman JC, Carrier L, al-Mahdawi S, et al (1997) The influence of the angiotensin I converting enzyme genotype in familial hypertrophic cardiomyopathy varies with the disease gene mutation. J Mol Cell Cardiol 29: 831–838. 9140839 10.1006/jmcc.1996.0332

[23] Kawaguchi H (2003) Angiotensin-converting enzyme and angiotensinogen gene polymorphism in hypertrophic cardiomyopathy. Exp Clin Cardiol 8: 155–159. 19641710 PMC2716279

[24] Ishanov A, Okamoto H, Yoneya K, Watanabe M, Nakagawa I, et al (1997) Angiotensinogen gene polymorphism in Japanese patients with hypertrophic cardiomyopathy. Am Heart J 133: 184–189. 9023164 10.1016/s0002-8703(97)70207-2

[25] Rai TS, Dhandapany PS, Ahluwalia TS, Bhardwaj M, Bahl A, et al (2008) ACE I/D polymorphism in Indian patients with hypertrophic cardiomyopathy and dilated cardiomyopathy. Mol Cell Biochem 311: 67–72. 18165925 10.1007/s11010-007-9695-z

[26] Osterop AP, Kofflard MJ, Sandkuijl LA, ten Cate FJ, Krams R, et al (1998) AT1 receptor A/C1166 polymorphism contributes to cardiac hypertrophy in subjects with hypertrophic cardiomyopathy. Hypertension 32: 825–830. 9822439 10.1161/01.hyp.32.5.825

[27] Thakkinstian A, McEvoy M, Chakravarthy U, Chakrabarti S, McKay GJ, et al (2012) The association between complement component 2/complement factor B polymorphisms and age-related macular degeneration: a HuGE review and meta-analysis. Am J Epidemiol 176: 361–372. 22869612 10.1093/aje/kws031 PMC6483268

[28] Thakkinstian A, McKay GJ, McEvoy M, Chakravarthy U, Chakrabarti S, et al (2011) Systematic review and meta-analysis of the association between complement component 3 and age-related macular degeneration: a HuGE review and meta-analysis. Am J Epidemiol 173: 1365–1379. 21576320 10.1093/aje/kwr025

[29] Luo R, Li X, Fan X, Yuan W, Wu X (2013) Association of Tumor Necrosis Factor-alpha Gene G-308A Polymorphism with Dilated Cardiomyopathy: A Meta-Analysis. DNA Cell Biol 32: 130–137. 23425028 10.1089/dna.2012.1911

[30] Thakkinstian A, McEvoy M, Minelli C, Gibson P, Hancox B, et al (2005) Systematic review and meta-analysis of the association between {beta}2-adrenoceptor polymorphisms and asthma: a HuGE review. Am J Epidemiol 162: 201–211. 15987731 10.1093/aje/kwi184

[31] Thakkinstian A, McElduff P, D'Este C, Duffy D, Attia J (2005) A method for meta-analysis of molecular association studies. Stat Med 24: 1291–1306. 15568190 10.1002/sim.2010

[32] Higgins JP, Thompson SG, Deeks JJ, Altman DG (2003) Measuring inconsistency in meta-analyses. Bmj 327: 557–560. 12958120 10.1136/bmj.327.7414.557 PMC192859

[33] Petitti DB (1994) Statistical methods in meta-analysis. In Meta-analysis, decision analysis, and cost-effectiveness analysis. DB Petitti, ed. (Oxford, New York), Chapter 7, pp. 90–114.

[34] Dupont WD, Plummer WD Jr (1990) Power and sample size calculations. A review and computer program. Control Clin Trials 11: 116–128. 2161310 10.1016/0197-2456(90)90005-m

[35] Coto E, Palacin M, Martin M, Castro MG, Reguero JR, et al (2010) Functional polymorphisms in genes of the Angiotensin and Serotonin systems and risk of hypertrophic cardiomyopathy: AT1R as a potential modifier. J Transl Med 8: 64. 20594303 10.1186/1479-5876-8-64 PMC2907326

[36] Kaya CT, Gurlek A, Altin T, Kilickap M, Karabulut HG, et al (2010) The relationship between angiotensin converting enzyme gene I/D polymorphism and QT dispersion in patients with hypertrophic cardiomyopathy. J Renin Angiotensin Aldosterone Syst 11: 192–197. 20478904 10.1177/1470320310368190

[37] Rao PPKM, Munshi A, Mullapudi R, Potham Sampath K, Sharath A, et al (2011) The M235T polymorphism of the angiotensinogen gene in South Indian patients of hypertrophic cardiomyopathy. J Renin Angiotensin Aldosterone Syst 12: 238–242. 21163864 10.1177/1470320310387955

[38] Brito D, Fernandes A, Bicho M, Madeira HC (2010) Functional variants of angiotensin-converting enzyme gene and phenotype expression in hypertrophic cardiomyopathy. European Journal of Heart Failure 12: S250–S251.

[39] Ogimoto A, Hamada M, Nakura J, Miki T, Hiwada K (2002) Relation between angiotensin-converting enzyme II genotype and atrial fibrillation in Japanese patients with hypertrophic cardiomyopathy. J Hum Genet 47: 184–189. 12166654 10.1007/s100380200021

[40] Doolan G, Nguyen L, Chung J, Ingles J, Semsarian C (2004) Progression of left ventricular hypertrophy and the angiotensin-converting enzyme gene polymorphism in hypertrophic cardiomyopathy. Int J Cardiol 96: 157–163. 15314809 10.1016/j.ijcard.2004.05.003

[41] Moiseev VS, Demurov LM, Kobalava Zh D, Chistiakov DA, Tereshchenko SN, et al (1997) [The polymorphism of the angiotensin-converting enzyme gene in patients with hypertension, left ventricular hypertrophy and the development of a myocardial infarct at a young age. Preliminary report]. Ter Arkh 69: 18–23. 9411819

[42] Cai SY, Shi YP, Yu F, Xu G (2004) [Association of angiotensinogen gene M235T variant with hypertrophic cardiomyopathy]. Zhonghua Yi Xue Yi Chuan Xue Za Zhi 21: 280–282. 15192838

[43] Kolder IC, Michels M, Christiaans I, Ten Cate FJ, Majoor-Krakauer D, et al The role of renin-angiotensin-aldosterone system polymorphisms in phenotypic expression of MYBPC3-related hypertrophic cardiomyopathy. Eur J Hum Genet 20: 1071–1077. 10.1038/ejhg.2012.48 PMC3449069 22569109

[44] Pfeufer A, Osterziel KJ, Urata H, Borck G, Schuster H, et al (1996) Angiotensin-converting enzyme and heart chymase gene polymorphisms in hypertrophic cardiomyopathy. Am J Cardiol 78: 362–364. 8759823 10.1016/s0002-9149(96)00296-2

[45] Schut AF, Bleumink GS, Stricker BH, Hofman A, Witteman JC, et al (2004) Angiotensin converting enzyme insertion/deletion polymorphism and the risk of heart failure in hypertensive subjects. Eur Heart J 25: 2143–2148. 15571830 10.1016/j.ehj.2004.08.026

[46] Malik FS, Lavie CJ, Mehra MR, Milani RV, Re RN (1997) Renin-angiotensin system: genes to bedside. Am Heart J 134: 514–526. 9327710 10.1016/s0002-8703(97)70089-9

[47] Timmermans PB, Duncia JV, Carini DJ, Chiu AT, Wong PC, et al (1995) Discovery of losartan, the first angiotensin II receptor antagonist. J Hum Hypertens 9 Suppl 5: S3–18. 8583479

[48] Dahlof B, Pennert K, Hansson L (1992) Reversal of left ventricular hypertrophy in hypertensive patients. A metaanalysis of 109 treatment studies. Am J Hypertens 5: 95–110. 1532319 10.1093/ajh/5.2.95

[49] Danser AH, Schalekamp MA, Bax WA, van den Brink AM, Saxena PR, et al (1995) Angiotensin-converting enzyme in the human heart. Effect of the deletion/insertion polymorphism. Circulation 92: 1387–1388. 7664416 10.1161/01.cir.92.6.1387

[50] Buck PC, Fernandes F, Arteaga E, Matsumoto AY, Araujo AQ, et al (2009) Association of angiotensin-converting enzyme activity and polymorphism with echocardiographic measures in familial and nonfamilial hypertrophic cardiomyopathy. Braz J Med Biol Res 42: 717–721. 19390744 10.1590/s0100-879x2009005000001

[51] Jeunemaitre X, Inoue I, Williams C, Charru A, Tichet J, et al (1997) Haplotypes of angiotensinogen in essential hypertension. Am J Hum Genet 60: 1448–1460. 9199566 10.1086/515452 PMC1716122

[52] Sethi AA, Nordestgaard BG, Tybjaerg-Hansen A (2003) Angiotensinogen gene polymorphism, plasma angiotensinogen, and risk of hypertension and ischemic heart disease: a meta-analysis. Arterioscler Thromb Vasc Biol 23: 1269–1275. 12805070 10.1161/01.ATV.0000079007.40884.5C

