# Supplementary material for: The Influence of Angiotensin Converting Enzyme and Angiotensinogen Gene Polymorphisms on Hypertrophic Cardiomyopathy
Source: PLoS One. 2013 Oct 25;8(10):e77030. doi: 10.1371/journal.pone.0077030 (PMC3808382; doi:10.1371/journal.pone.0077030)
Supplement: Appendix S1 — An appendix for Stata commands used in the present meta-analysis. (DOC) [file pone.0077030.s002.doc]

An appendix for Stata commands used in the present meta-analysis

| Commands | Role |
| --- | --- |
| genhwcci | to test Hardy-Weinberg Equilibrium |
| metagen | to determine generic model |
| xtlogit, lrtest, | to calculate overall gene effect |
| metan | Meta-analysis for OR and standard mean difference |
| metareg | Meta-regression for source of heterogeneity |
| metabias | Egger’s test for publication bias |
| metaninf | Sensitivity analysis |
